# Bilateral Luxatio Erecta Humeri

**DOI:** 10.5811/cpcem.2021.1.51255

**Published:** 2021-03-01

**Authors:** Miguel A. Martinez-Romo, Shahram Lotfipour, C. Eric McCoy

**Affiliations:** University of California, Irvine Medical Center, Department of Emergency Medicine, Orange, California

**Keywords:** Luxatio erecta, bilateral luxatio erecta, luxatio erecta humeri, shoulder dislocation, inferior glenohumeral dislocation

## Abstract

**Case Presentation:**

We describe a middle-aged male presenting to the emergency department with bilateral shoulder pain, holding both arms in abduction after trauma. Radiographs demonstrated a bilateral inferior dislocation of the glenohumeral joints consistent with luxatio erecta humeri.

**Discussion:**

We review the clinical presentation of luxatio erecta and its complications. We also describe the characteristic presentation on radiographs. Our case illustrates the hallmark findings of luxatio erecta of an abducted humeral shaft parallel to the scapular spine.

## CASE PRESENTATION

**Image 1:**
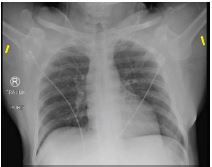
The patient presented with his humeri in fixed abduction (yellow arrows).

**Image 2:**
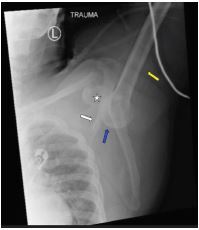
Left shoulder radiograph demonstrating the shaft of the humerus (yellow arrow) in fixed abduction. The humeral head (blue arrow) is inferior to the glenoid fossa (star). Note the scapular spine (white arrow) is almost parallel to the shaft of the humerus (yellow arrow).

**Image 3:**
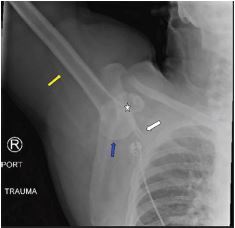
Right shoulder radiograph demonstrating the shaft of the humerus (yellow arrow) in fixed abduction. The humeral head (blue arrow) is inferior to the glenoid fossa (star). Note the scapular spine (white arrow) is almost parallel to the shaft of the humerus (yellow arrow).

A 53-year-old male with a history of diabetes presented to the emergency department (ED) with bilateral shoulder pain. He was the victim of a carjacking; he held on to his car as the perpetrator sped away, was subsequently dragged, and then run over by the vehicle. He presented with both arms fully abducted and fixed. Imaging revealed bilateral inferior dislocation of the glenohumeral joint consistent with bilateral luxatio erecta, which were reduced in the ED (Images 1–3).

## DISCUSSION

Luxatio erecta humeri is the inferior dislocation of the glenohumeral joint.^1^,^2^ Luxatio erecta makes up 0.5% of all shoulder dislocations, making a bilateral presentation even more rare.^1^,^2^ By comparison, anterior dislocations make-up 95–97% of dislocations, while posterior dislocations make up 2–4% of all dislocations.^1^ Luxatio erecta can happen in any age group, infants to elderly, with the classic presentation being a person who presents with fixed hyperabduction of the arm at the shoulder, flexion at the elbow, and pronation of the forearm.^1^,^2^ The mechanism of injury is a direct loading force on a full abducted arm or a sudden hyperabduction of an abducted arm.^2^

Radiographs will demonstrate the humeral heads at the subglenoid region, with an abducted humeral shaft almost parallel to the scapular spine.^3^ Treatment involves reduction of the joint, which can be achieved by performing procedural sedation followed by the traction-countertraction technique.^1^–^4^ Thereafter, the patient’s arm is put into a sling in full adduction for immobilization, and 1–2 week orthopedic follow-up should be arranged.^1^,^2^,^4^ Complications include avulsed shoulder capsule, torn rotator cuff tendons or injury to adjacent muscles, fractures of the acromion, clavicle, inferior glenoid fossa, or greater tuberosity, brachial plexus injuries, and axillary vessel injury.^1^–^3^

**CPC-EM Capsule**

What do we already know about this clinical entity?

*Luxatio erecta humeri is a rare presentation of a shoulder dislocation, with a bilateral presentation being even more rare.*

What is the major impact of the image(s)?

*Patients have a hyperabducted arm, imaging reveals a subglenoid humeral head, and treatment involves reduction.*

How might this improve emergency medicine practice?

*Proper recognition allows for reduction, immobilization, and arrangement of Orthopedic follow-up, all of which can potentially reduce long-term sequelae.*
